# Leveraging Synthetic Data to Facilitate Research: A Collaborative Model for Analyzing Sensitive National Cancer Registry Data in England

**DOI:** 10.1007/s43441-025-00820-z

**Published:** 2025-06-05

**Authors:** George Kafatos, Julia Levy, Sophie Jose, Pooja Hindocha, Olia Archangelidi, Sally Vernon, Lora Frayling

**Affiliations:** 1 Center for Observational Research, Amgen Ltd, Uxbridge, UK; 2 IQVIA Ltd, London, UK; 3 Health Data Insight CIC, Cambridge, UK; 4 National Disease Registration Service England, London, UK

**Keywords:** Real-world data, Cancer research, Synthetic data, Cancer analysis system, Simulacrum

## Abstract

Real-world data (RWD) are increasingly recognized as critical to advancing drug development and health care delivery, with regulatory bodies increasingly recognising their value. However, stringent governance requirements, while essential for protecting patient privacy, create significant challenges for conducting research. The Cancer Analysis System (CAS), managed by National Health Service (NHS) England, includes a national cancer registry and linked health care datasets. To address data access challenges, Simulacrum, a set of publicly available synthetic datasets generated from the CAS, can be used to carry out preliminary data analysis, hypothesis generation and development of programming code that can be executed to run analyses on CAS data. This paper presents a collaborative operating model that leverages Simulacrum to enable efficient, privacy-compliant analytics. Analysis of 18 projects conducted using this model demonstrated an average duration of 2.3 months from the start of Code Development to Data Release (CDDR). By enabling researchers to conduct privacy-compliant analysis on synthetic data, this approach increases transparency by providing insights into patient-level data while reduces reliance on custodians of sensitive data. Our findings highlight how synthetic data can be leveraged to facilitate efficient research on restricted patient-level RWD, while safeguarding patient privacy. This framework offers a scalable solution for other data custodians that can enable broader use of RWD, accelerating healthcare innovation.

## Introduction

Real-world data (RWD) are increasingly recognized as playing an integral role in advancing drug development and understanding health care delivery [1]. By offering valuable insights into disease causes and outcomes, identifying drug targets for precision medicine, and supporting disease prediction and prevention, RWD play a pivotal role in improving patient health outcomes [2]. Regulatory and reimbursement authorities have recognized the importance of RWD for evidence-based decision making. For instance, the UK National Institute for health and Care Excellence (NICE) in its 2022 Real-World Evidence Framework highlighted that “Real-world data can improve our understanding of health and social care delivery, patient health and experiences, and the effects of interventions on patient and system outcomes in routine settings” [3].

**Fig. 1:**
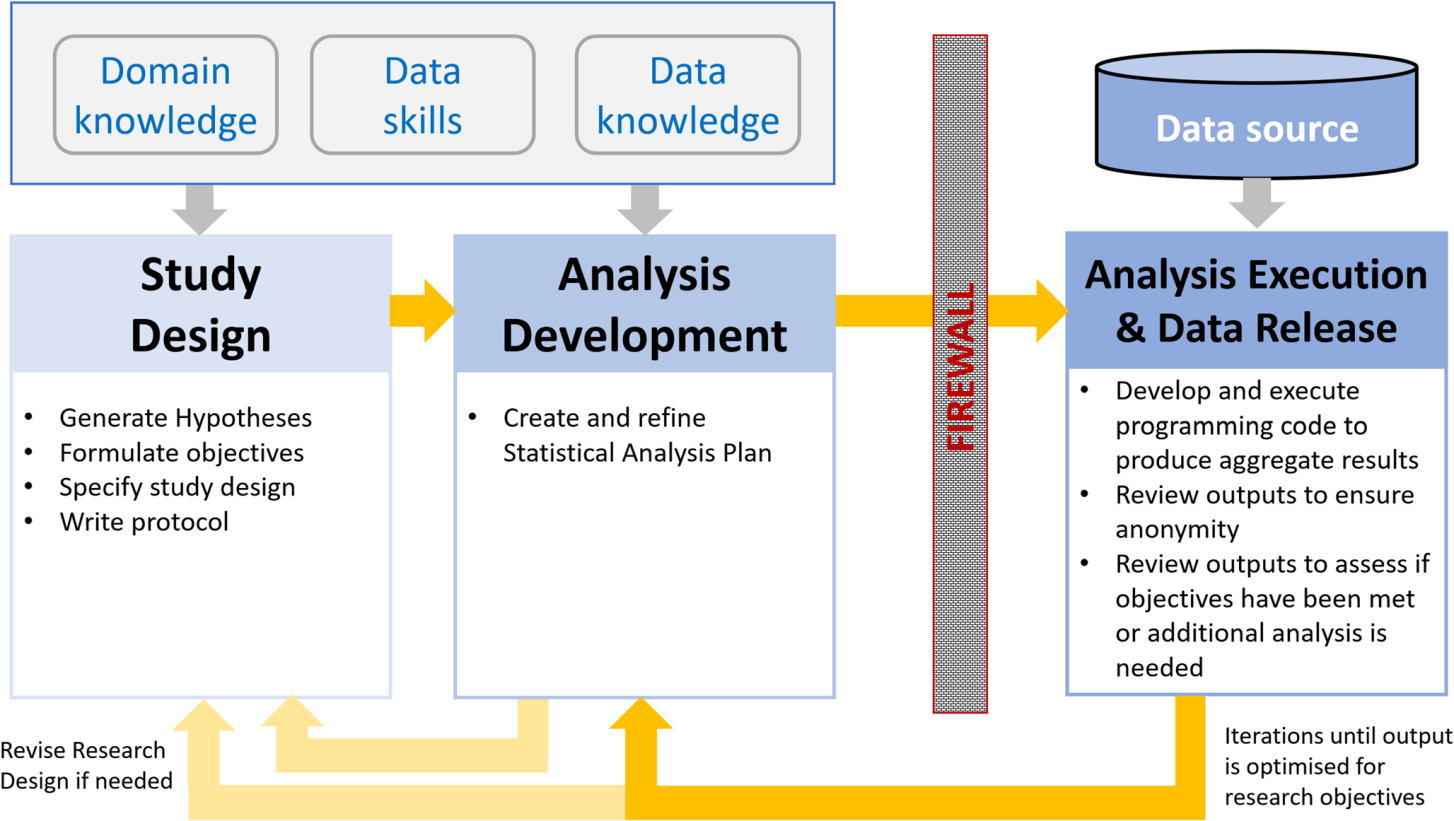
Illustration of the typical workflow for research with sensitive data. Study Design and Analysis Development are informed by the researchers’ domain knowledge, data skills and knowledge of the data source prior to the Analysis Execution to produce aggregate data outputs for Data Release. Analysis Development process may be repeated to optimise outputs

The growing impact of RWD can be attributed to the increased acceptance of regulatory and reimbursement bodies as well as clinical decision makers [4]. This has been enabled by the digitalization of health services and advancements in computing and health informatics, which have expanded the availability of data sources [4, 5]. However, maintaining the privacy of individuals’ personal health data remains, justifiably so, a critical concern. Legal obligations such as the European Union’s General Data Protection Regulation (GDPR) regulate how personally identifiable data (defined as any information that can directly or indirectly identify an individual) are collected, stored and used [9, 10]. These restrictions often limit researchers’ access to patient-level data, creating challenges for conducting timely and efficient research. In regions with stringent privacy regulations, sensitive data are often accessible only to data custodians or Sensitive Health Data (SHD) analysts with data access privileges who are trained in responsible data use. Researchers who are unable to get data access need to design their studies with limited insights from the data and rely heavily on the SHD analysts to conduct different stages of research. The different stages of research include [1] generating hypotheses, study design, protocol (“Study Design” stage) [2], Statistical Analysis Plan (SAP) development (“Analysis Development” stage) and [3] analysis on the patient-level data that can only be executed by the SHD analysts (“Analysis Execution & Data Release” stage). Analysis execution is performed on the data source in a secure environment protected by a firewall (Fig. 1). This can be an iterative and resource-intensive process. Furthermore, due to the high demand for data-supported research, the capacity of SHD analysts is often insufficient, leading to delays in conducting research.

To address these challenges, anonymization methods, that typically involve removing or modifying direct and indirect patient identifiers, are commonly used [6]. While these methods protect privacy, they can significantly compromise data utility, especially when demographic characteristics that are critical for analyses are omitted. Similarly, aggregate-level data, such as the statistical outputs provided by National Health System (NHS) England’s National Disease Registration Service (NDRS), offer privacy protection but often lack the granularity needed for answering specific research questions [7]. An alternative approach that has recently emerged involves the use of synthetic data, i.e., artificial datasets generated to mimic the structure and characteristics of real data [8–10]. Synthetic data allow researchers to conduct initial feasibility analysis (such as generating hypotheses, determining the sample size, assessing data completeness and exploring different methodological approaches) and to develop programming code, while safeguarding patient privacy [11]. This approach optimizes the research process as the programming code can be available before transitioning to analysis on real data.

Despite its potential, there are limited publications demonstrating how synthetic data can be leveraged to facilitate research on restricted patient-level data. This paper addresses this gap by describing a collaborative operating model that leverages Simulacrum, a synthetic version of the Cancer Analysis System (CAS). The CAS is a comprehensive database that includes cancer patient registrations in England and linked health care databases and is managed by the NDRS, part of NHS England [12]. The NDRS facilitates analysis to CAS data while ensuring robust patient data protection, in partnership with organizations such as Health Data Insight (HDI) who employ SHD analysts. HDI, with support from IQVIA and AstraZeneca, has developed Simulacrum, a synthetic version of the CAS designed to provide data insights without compromising patient privacy [12]. This paper describes a collaborative operating model that leverages Simulacrum synthetic data to enable transparent, efficient and privacy-compliant analytics.

## Materials and Methods

### The CAS Database

The CAS contains information pertaining to over 98% of individuals diagnosed and treated with cancer in England [13]. It is collated and maintained by the NDRS [12]. It integrates information from several data sources such as [1] patient and tumour information from the National Cancer Registration Dataset (NCRD) [2], treatment information (such as chemotherapy, immunotherapy and radiotherapy treatment) from the Systemic Anti-Cancer Therapy Dataset (SACT) and the National Radiotherapy Dataset (RTDS) [3], hospital admissions and surgeries from the Hospital Episode Statistics (HES) [4] mortality data from the Office of National Statistics (ONS) [12, 14–18] and [5] biomarker testing data from the somatic molecular diagnostic testing dataset (CAS-MDx), which was added more recently [14].

### The Simulacrum Synthetic Dataset

Simulacrum is a synthetic database that closely resembles datasets in the CAS without including any patient-identifiable data. It maintains a similar data structure while preserving statistical characteristics of the CAS data, including marginal distributions, key multivariate distributions and temporal characteristics. Simulacrum ensures alignment of high-level counts between the real CAS and the synthetic data [19]. A Bayesian network approach was employed to generate the synthetic patient-level data while protecting patient privacy [20, 21]. Simulacrum includes synthetic versions of the NCRD, SACT, RTDS and CAS-MDx datasets (the HES information is not included) along with most of their available data variables [22].

### Collaborative Parties and Data Access Governance

The NDRS, as the data custodian, has established a formal partnership with HDI, a specialist in cancer patient data, under a structured governance framework (https://digital.nhs.uk/ndrs/our-work/ncras-partnerships/health-data-insight). HDI, in its role as an SHD analyst, collaborates with IQVIA, a healthcare insights provider, to support observational research (OR) groups in conducting studies using CAS data. This partnership operates within an official and transparent data access mechanism, ensuring that access is granted through a governed and equitable process. This paper focuses on one OR group that participated in this established model: the Amgen Center for Observational Research (CfOR).

### Collaborative Operating Model

The collaborative operating model has been approved by NHSE. It enables the OR group to conduct full analytic projects using CAS data, with support from the SHD analysts and HI provider. Each analytic project typically aims to answer predefined research questions, which must offer benefit to patients, for example by providing novel insights into disease management and outcomes, highlighting areas of unmet need or supporting clinical trial assessments. Analytic projects are often related, exploring the same research topics and having related research questions.

**Fig. 2:**
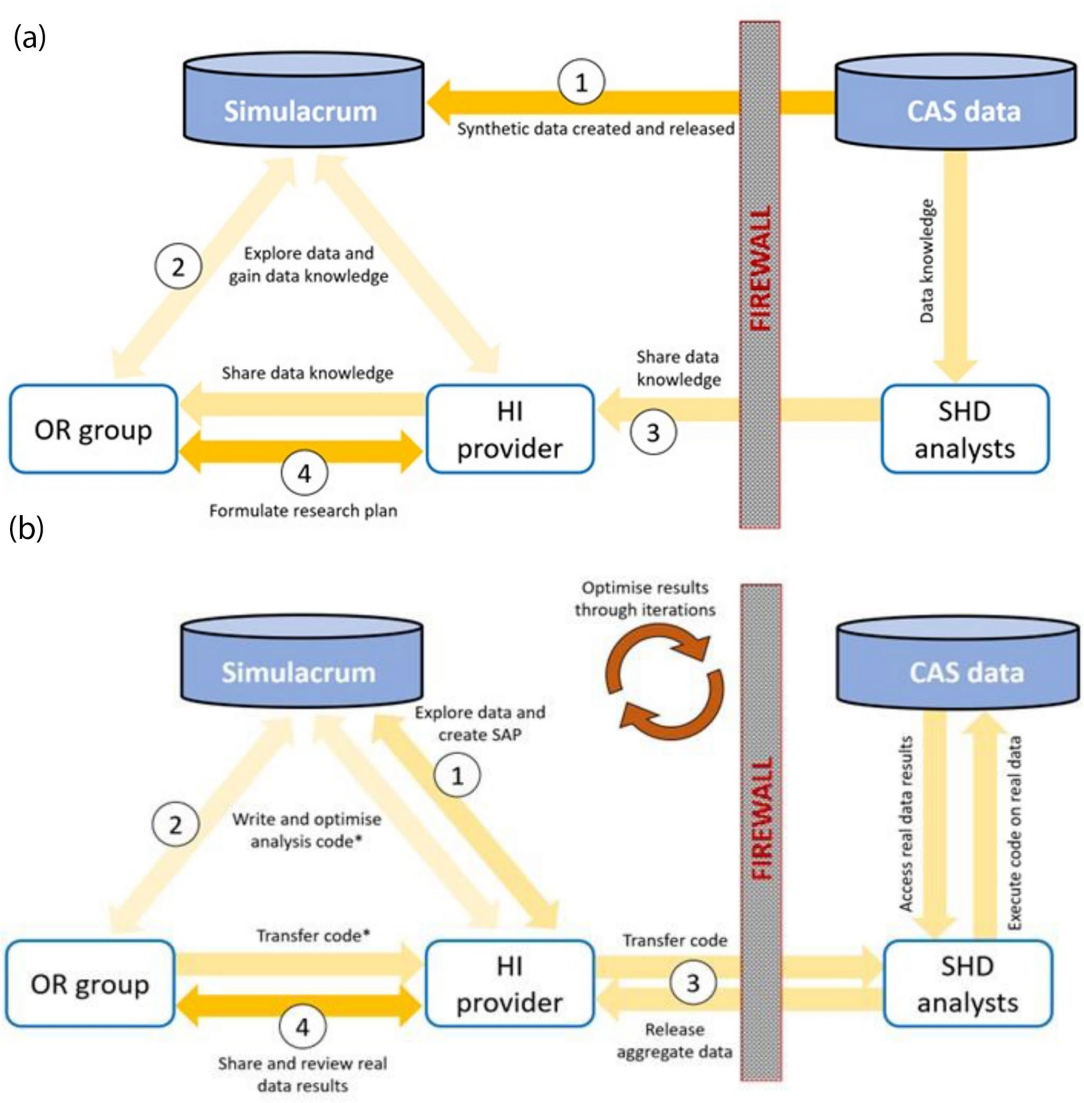
Collaboration model leveraging Simulacrum synthetic data. **(a)** Study Design stage, (1) Simulacrum is created and released based on the real CAS data. (2) The OR group and HI provider explore the Simulacrum data to gain understanding of CAS. (3) SHD analysts share their knowledge about the data source with the HI provider who, in turn, shares with the OR groups. (4) The OR group and HI provider work together to formulate the research plan based on their data knowledge and domain expertise. **(b)** Analysis Development and Data Release stage, (1) The HI provider creates the SAP informed by the Study Design, knowledge of the data and their expertise. (2) The HI provider* uses the synthetic data to write, test and optimise the programming code. (3) This code is transferred to the SHD analysts who run it on the real data and release aggregate data results to the HI provider after applying privacy checks. (4) The HI provider shares the results with the OR group and they review them to ensure they achieve the research objectives. If not, the process is iteratively repeated to optimise the finalised data results. * The OR Group develops programming code in some cases for simple projects

The workflow of the collaborative operating model includes three stages:

1. *Study Design*: The OR group formulates objectives based on their research questions, domain knowledge, and often by leveraging Simulacrum to explore the data. They collaborate with the HI provider to outline study parameters (such as population, study period and outcomes) and establish the patient eligibility criteria and analysis types. HI analysts advise on database characteristics and feasibility, often incorporating input from SHD analysts regarding sensitive data and cohort considerations. The use of Simulacrum and other insights based on experience working with the data allow the OR group and HI provider to refine study designs independently, minimizing reliance on SHD analysts (Fig. 2a).

2. *Analysis Development*: HI experts collaborate with the OR group to develop the SAP. The SAP defines study cohort criteria, lists study variables, and describes data analysis and anticipated outputs. Programming code is developed and tested using the Simulacrum data, given these approximate the CAS database structure and statistical properties. This preparatory work ensures robust study designs and analysis plans before applying them to sensitive CAS data. If new insights emerge or adjustments are needed, further refinement of the code may be required (Fig. 2b).
3. *Analysis Execution and Data Release*: SHD analysts execute the refined programming code on CAS data during code-running sessions, producing aggregate-level outputs. During these sessions, HI experts, that can include programmers, statisticians, epidemiologists and clinicians, review the outputs alongside SHD analysts to ensure the code runs as intended and aligns with study objectives. OR researchers may also participate to validate results against the study design (Fig. 2b). All sessions are conducted with stringent privacy protections, ensuring that neither HI experts nor OR researchers see any identifiable patient data. The aggregate-level outputs are subjected to privacy assessment and anonymization prior to release, including low-resolution masking for interim analyses and high-resolution masking for final data releases. Masking involves rounding and suppression of aggregate patient numbers (Table 1). Results are reviewed by SHD analysts and approved by the NDRS Caldicott Guardian before release. Once released HI and OR researchers review the results to check they fulfil the study aims. If required, further iterations are conducted to refine the analysis based on anonymized outputs (Fig. 2b). Once study aims are fulfilled, the project is concluded.

**Table 1:** Rules for releasing “masked” output to protect patient privacy

Masking level	Description
Low resolution	• All small numbers 1–9 are suppressed and replaced with *
	• All numbers above 10 are rounded to the nearest 10
	• Percentages are rounded to the nearest 5%
	• Minimum and Maximum are replaced with 5th and 95th percentile
High resolution	• All small numbers 1–5 are suppressed and replaced with *
	• Minimum and Maximum are replaced with 5th and 95th percentile

### Developing Metrics for Assessing the Collaborative Model

To evaluate the collaborative operating model, information was collected from 18 analytic projects (exploring 12 research topics) conducted by the OR group, between 2021 and 2024. The assessment focused on project timelines, iteration frequencies, and common practices, particularly in the final two research stages: Analysis Development and Analysis Execution & Data Release.

Key timeline dates were defined from data related to projects and operations. These data included:

- Code-running sessions and data release information were inferred from multiple resources, including logs of data releases, file storage of released data, emails and digital tools used to schedule and coordinate sessions.
- Key dates and information about projects were inferred, where possible, from project agreement forms between SHD and HI teams that approve the start of Analysis Development (AD) and estimate project timelines. When specific dates were missing, they were estimated based on the following rules:
  - AD start date: Assigned based on project’s AD approval date. If missing due to dependence on a previous project, it was set to 14 days after the previous project’s final data release. Note that all time durations expressed in “days” refer to calendar days.
  - Project end date: Determined from the agreed project completion data. If missing, it was set to 14 days after the final data release.
  - Programming code development start date: Taken from the planned code development start date. If missing, the project was excluded from code development-related metrics.
- Information about specific analysis aspects, e.g., the type of cohort, the datasets used in analysis and the type of analysis performed was collected by inspecting programming code and data release outputs.

These were used to derive two key metrics that describe project timelines:

- Analysis Development and Data Release (ADDR) time: The time duration between Analysis Development start date and the final data release before the project end date.
- Code Development and Data Release (CDDR) time: The time duration between the programming code development start date and the final data release before the project end date.

## Results

### Project Duration

**Fig. 3:**
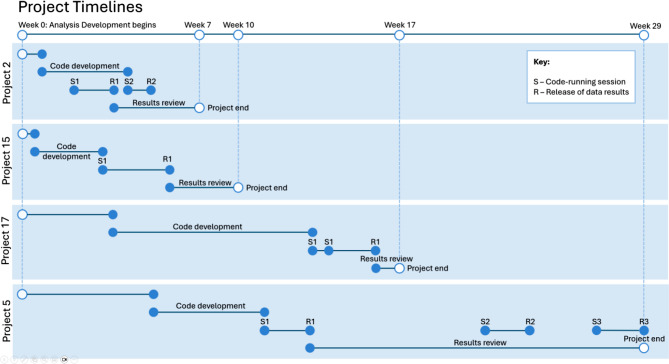
Timelines of four projects

The 18 analytic projects were summarised based on timelines and efficiency metrics (Table 2). The CDDR time ranged from 0.7 months (22 days) to a maximum of 5.4 months, with an average duration of 2.3 months across analytic projects. The average ADDR time was slightly longer at 3 months, with the majority of the projects (10 out of 18) obtaining the final data release within 3 months of AD start. However, some projects required longer durations, with the maximum recorded ADDR time being 7.8 months. For the 9 projects that had independent research topics, the average CDDR and ADDR time were significantly shorter at 1.5 and 2.0 months, respectively. Figure 3 illustrates timeline variability across four projects, highlighting differences in duration, the number of visits, and intervals between key milestones.

**Table 2:** Information about 18 projects conducted using collaborative operating model

Analytic project id	Patient Cohort	Research topic ID	Number of data releases	ADDR time (months)	CDDR time (months)	% of ADDR time spent in CDDR
P1	Metastatic Prostate Cancer	R1	1	2.9	1.6	56
P2	Acute Lymphoblastic Leukemia	R2	2	1.2	1.1	86
P3	Metastatic Castration-Resistant Prostate Cancer	R3	1	1.5	-	-
P4	Acute Lymphoblastic Leukemia	R4	1	3.3	-	-
P5	Acute Lymphoblastic Leukemia	R4	3	6.7	5.2	78
P6	Myelosuppressive Therapy	R5	1	1.0	-	-
P7	Multiple Myeloma	R6	1	3.1	-	-
P8	Prostate Cancer	R7	1	2.2	-	-
P9	Non-Small Cell Lung Cancer	R8	1	3.9	0.7	17
P10	Gastric Cancer	R9	1	2.3	1.6	71
P11	Gastric Cancer	R9	2	7.8	5.4	67
P12	Gastric Cancer	R9	2	5.7	3.9	68
P13	Small Cell Lung Cancer	R10	1	0.9	0.7	75
P14	Small Cell Lung Cancer	R10	2	3.4	3.5	98
P15	Small Cell Lung Cancer	R10	1	3.5	2.6	75
P16	Small Cell Lung Cancer	R10	2	2.6	1.4	54
P17	Metastatic Prostate Cancer	R11	1	1.6	1.5	94
P18	Ewing Sarcoma	R12	1	1.1	0.9	79

- Start date of code development is missing

To understand how much of ADDR time was spent in code development and data release iterations compared to other research activities we look at the percentage of ADDR spent in CDDR for projects that had a valid programming start date. Approximately 71% of the ADDR time was dedicated to the CDDR stage across projects, while the remaining 29% was spent on other aspects of AD, including the development of Statistical Analysis Plans (SAPs). However, this percentage varied significantly across projects, between 17 and 98%, which suggests that multiple factors influenced project durations.

### Analysis Execution

Code-running sessions were typically scheduled for 2 to 8 h of SHD analysts’ time, occasionally split over two days. On average, these sessions lasted 5 h per project. Additional time was spent on preparatory activities, including discussions between SHD and HI analysts and the extraction of data in advance of the sessions (Table 3).

**Table 3:** Project statistics

Parameter	Estimate
Total number of projects	*n* = 18
Time spent in ADDR (months)	mean = 3.0; range: 0.9, 7.8
Time in CDDR (months)	mean = 2.3; range: 0.7, 5.4
Percentage of ADDR spent in CDDR*	mean = 71%; range: 17%, 98%
Total number of code-running sessions	*n* = 25
Number of sessions per project	mean = 1.4
Total number of code-running session days	*n* = 32
Time spent in code-running sessions per project (hours)**	mean = 5
Total number of data releases	25
Time from request to data release (days)	mean = 13.8; range: 3, 22

*Based on the 13 projects where CD start date is not missing; ** assume 4 h per session on a day

Notably, 12 (out of 18) analytic projects were completed with a single code-running session and data release, requiring no additional iterations. For projects requiring iterations, the maximum observed was three. The need for iterations arose due to:

- Further refinement of analysis based on intermediary data outputs, such as adjustments to treatment definitions (e.g., lines of therapy [LOT]).
- Detection of programming code errors during or after code-running sessions.
- Modifications to the presentation of results, such as adjustments to graphs.

### Data Releases

The collaborative model facilitated rapid release of anonymized aggregate data. The average time from a data release request to delivery was 14 days, with turnaround times ranging from as little as 7 days (for expedited requests) to a maximum of 22 days (due to complex privacy requirements or reduced capacity during holidays). These efficiencies were supported by the implementation of masking rules in the analysis code by the HI provider.

### Types of Analysis Projects

**Fig. 4:**
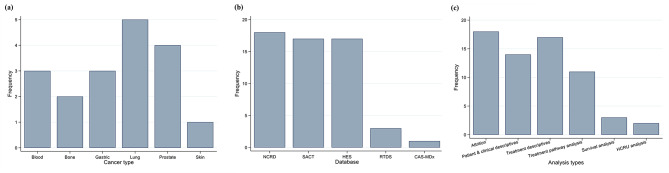
Distributions by cancer types, data sources and analysis types across 18 projects that were executed as part of the collaborative operating model. (**a**) Cancer types. (**b**) Data sources, Note: For most projects a combination of these data sources was used. The CAS MDx and RTDS were added to the data set more recently. (**c**) Analysis types. Note: HCRU stands or Healthcare Resource Utilisation

The collaborative model supported a wide range of research projects across different cancer cohorts. Multiple projects involved similar cancer types (e.g., prostate cancer, blood cancer, lung cancer), similar datasets and comparable analysis plans which allowed the development of dataset and domain-specific expertise within the HI and OR teams (Fig. 4).

## Discussion

In recent years, there has been a growing body of peer-reviewed literature in the health care sector focusing on synthetic data generation and evaluation, highlighting its potential to enhance access to RWD while preserving patient privacy [11, 23–25]. However, there are limited examples of peer-reviewed publications demonstrating how synthetic data can be used in practice. This paper addresses this gap by describing a novel collaborative operational model that leverages synthetic data to enable transparent, efficient and privacy-compliant analysis of the CAS data in England.

The incorporation of Simulacrum synthetic data reduces the dependence of the OR group and HI provider on SHD analysts across the project. Since the Simulacrum retains similar structural and statistical properties to the CAS, it is possible to formulate hypotheses, specify study protocols and develop programming code independently from SHD analysts. Reliance on the SHD analysts is instead concentrated on a small number of code-running sessions and data release. This enables research that is not otherwise possible in cases where researchers cannot get access the data and capacity of SHD analysts is limited.

The statistical similarities of Simulacrum data enable a close alignment of high-level descriptive statistics for general cancer cohorts with those of the real data. However, for more specialised cohorts and complex analyses that involve many data variables across tables, the results derived from Simulacrum may be less reflective of the real data. In our experience, while Simulacrum closely replicates key summary statistics and marginal distributions, results may diverge more noticeably from real data when analyzing groups of patients defined by a broader set of characteristics. For example, when counting patients who have a specific cancer type, stage, age and gender, discrepancies between synthetic and real CAS data outputs are expected to be broader than when just counting patients with a specific cancer site. These larger deviations are likely due to the inherent difficulty in preserving high-dimensional joint distributions in synthetic data generation. Furthermore, over time the discrepancies between the real and synthetic data increase, as the real data is updated over time. This can limit the insight researchers can gain to inform study design and SAP formulation, while making it more difficult to write code that can run on the real data without errors and corrections. For these reasons, it is not designed for making epidemiological inferences or clinical decisions [22]. Instead, Simulacrum can be used for hypothesis generation, defining the scope of analyses during preliminary research phases and to write analytical programming code that can be executed on the real data. Although such code can be easily transferrable to the CAS data, some errors may still arise when running it on the real data because of the statistical differences from real data. A formal evaluation of Simulacrum fidelity, privacy risk, and utility compared to the real CAS data is currently underway. The findings from this validation effort will be reported separately in a dedicated publication.

The collaborative operating model has also allowed the development of database- and domain-specific expertise within the HI provider and the OR group, as projects often focused on the same cancer types and using the same datasets. Although operational improvements and efficiencies were gained over the 3-year study period, particularly as the OR group became more familiar with the data, the overall project timelines did not appear to improve over time. This is likely due to the variation in complexity of the projects, a likely strong factor in determining project length. This accumulated knowledge streamlined project execution by further minimizing the need for input from SHD analysts. Similarly, the HI provider and SHD analysts were able to build an expertise and knowledge base from consistent use of both the synthetic and real patient data. For most of the projects, the data were released after a single code-running session, demonstrating both the quality of programming code developed using the Simulacrum and the ability of HI experts and SHD analysts to predict and swiftly resolve issues during code-running sessions. Such issues are often because Simulacrum doesn’t perfectly capture all CAS statistical properties and therefore cannot be a perfect substitute for CAS to test code. In practice, several factors contributed to variation in the proportion of ADDR time dedicated to CDDR and hence, the overall project duration across the 18 studies. These included differences in research project complexity, the number of required analytic iterations following interim data reviews and coordinating availability of SHD and HI analysts. To fasten the delivery of the projects, the following strategies were included: reuse of programming code from similar prior projects, early development and refinement of SAPs using the Simulacrum synthetic dataset, and proactive coordination with SHD analysts to align on timelines. One limitation of this study is that we have not accounted for research complexity, due to difficulty in quantifying this, and thus cannot reflect on it’s impact on timelines compared to other factors.

This paper focuses on the operating model and involvement of a single OR group, however there are some differences with how other OR groups might work with the CAS data. In particular, other OR groups are not typically actively involved with using the Simulacrum data directly for supporting the Study Design or Analysis Development stages. The OR group involved in the collaborative operating model described herein used Simulacrum to support hypotheses generation and feasibility analysis and in some, less complex cases, developing programming code, with close collaboration between the OR group and HI provider throughout the different stages of the analysis.

While this study demonstrates the benefits of a collaborative model leveraging synthetic data, we acknowledge that a direct comparison with alternative data governance models, such as those that do not use synthetic data, could offer additional insights. However, such a comparison is challenging due to the differences between data governance requirements and data access processes. In addition, there is limited published information in reporting timelines for data access and analysis, meaning there is lack of available statistics for comparison. As such, this paper provides helpful metrics and statistics that can be used to benchmark research timelines for other data sources.

## Conclusion

This paper describes a collaborative operating model designed to accelerate health care innovation by enabling efficient research projects that involve multiple expert organisations. A key component of this model is the use of synthetic data, generated from the original health data, to facilitate the different research stages. By studying 18 projects that involve access to the CAS patient data, we demonstrate that the approach leads to well-established and effective research practices. The collaborative operating model demonstrates the value of synthetic data in facilitating efficient research while maintaining privacy compliance. This framework offers a scalable solution for other data custodians, enabling broader use of RWD and advancing healthcare innovation.

## Data Availability

No datasets were generated or analysed during the current study.

## Declarations

### Target Journal

Therapeutic Innovation & Regulatory Science.

### Type of Publication

Analytical Report.
